# Sustainable Composites from Recycled Polypropylene and Hazelnut Shell Flour for Application in Irrigation Systems

**DOI:** 10.3390/polym17233207

**Published:** 2025-12-01

**Authors:** Francesco Paolo La Mantia, Roberto Scaffaro, Giuseppe Balsamo, Carmelo Giuffré, Erica Gea Rodi, Simone Corviseri, Maria Clara Citarrella

**Affiliations:** 1 INSTM—Consortium for Materials Science and Technology, Via Giusti 9, 50125 Florence, FI, Italy; giuseppe.balsamo01@communityunipa.it (G.B.); mariaclara.citarrella@unipa.it (M.C.C.); 2 Department of Engineering, University of Palermo, Viale Delle Scienze, ed. 6, 90128 Palermo, PA, Italy; 3 Irritec S.p.A., Via Gambitta Conforto, 98071 Capo d’Orlando, ME, Italy; carmelo.giuffre@irritec.com (C.G.); erica.rodi@irritec.com (E.G.R.); simone.corviseri@irritec.com (S.C.)

**Keywords:** recycled polypropylene, hazelnut shell, agro-industrial waste, natural fillers, composites, circular economy, sustainable agriculture, rheological properties, mechanical properties, irrigation fittings

## Abstract

The irrigation sector urgently needs more eco-sustainable materials able to guarantee the same performance as traditional fittings manufactured from virgin fossil-based polymers. In this study, sustainable composites were developed by melt-compounding virgin and recycled polypropylene (RPP) with hazelnut shell (HS) powder with or without maleic-anhydride-grafted polypropylene (PPC) coupling agent. The materials were characterized by a rheological and mechanical point of view. At high shear rates, the viscosity curves of matrices and composites converge, making the difference between neat and filled systems negligible in terms of processability. This indicates that standard injection-molding parameters used for the neat matrices can also be applied to the composites without significant adjustments. Tensile tests showed that adding 10 wt% HS powder increased the elastic modulus by approximately 30% (from 960 MPa to 1.2 GPa) while reducing elongation at break by about 90% compared with neat RPP. The use of PPC mitigated this loss of ductility, partially restoring tensile strength and increasing EB from 6% to 18% in RPP-based composites (+200%). Finally, sleeve bodies and nuts injection-molded from RPP/HS5 and RPP/HS5/PPC successfully resisted internal water pressure up to 3.5 bar without leakage or structural damage. These findings demonstrate that agro-industrial waste can be effectively valorized as a functional filler in recycled polypropylene, enabling the manufacture of irrigation fittings with mechanical and processing performances comparable to those of virgin PP and supporting the transition toward a circular economy.

## 1. Introduction

The transition to a circular economy requires strategies that simultaneously reduce the consumption of fossil resources and limit the accumulation of post-consumer waste. Mechanical recycling of plastics is one of the most effective approaches to achieve these goals, because it decreases the demand of virgin raw materials and lowers the overall carbon footprint of polymer production [1]. Moreover, for polyolefins such as polypropylene (PP), recycling reduces the need for petroleum-derived feedstocks and therefore mitigates CO_2_ emissions [2]. However, despite these advantages, the reuse of polypropylene is not free from challenges. During service life and throughout successive processing cycles, PP chains undergo severe thermo-oxidative degradation, chain scission, and structural rearrangements that lead to a progressive loss of molecular weight and a deterioration of mechanical and rheological properties [3,4]. Recycled polypropylene (RPP) often exhibits reduced ductility, lower impact strength, and a viscosity profile that can compromise its processability. For these reasons, RPP is commonly used in applications with modest performance requirements or is blended in limited amounts—typically below 30 wt%—with virgin PP in so-called “monopolymer blends” to mitigate the defects and maintain acceptable properties [5,6].

A promising strategy to enhance the performance of recycled plastics and, at the same time, to increase further the valorization of waste material is the incorporation of natural fillers [7,8,9]. In general, the use of bio-fillers offers multiple synergistic advantages: (i) it allows the inclusion of a higher fraction of recycled polymer while maintaining adequate mechanical properties; (ii) it introduces biodegradable/compostable and renewable materials into the formulation; (iii) it valorizes agricultural or agro-industrial residues that would otherwise require disposal; (iv) it can reduce the overall production costs [10,11,12,13,14,15,16]. In more detail, different lignocellulosic fillers have been widely investigated as reinforcing agents for polymeric and biopolymeric matrices [17,18,19,20,21,22]. In recent years, considerable attention has also been dedicated to the development of bio-reinforced composites that combine recycled polymers with natural fillers to couple mechanical performance with environmental sustainability. The use of waste-derived lignocellulosic materials as reinforcing agents addresses two key environmental issues simultaneously: the management of post-consumer plastics and the valorization of agro-industrial by-products [23,24]. Numerous studies have shown that incorporating wood flour, rice husk, nutshell powders, or other agricultural residues into recycled polyolefin matrices can increase stiffness, reduce material costs, and confer a reduction in the overall environmental footprint of the final product [18,25,26,27,28]. Among the wide variety of agricultural residues, hazelnut shells are generated in large quantities by the food industry and are still under-exploited as secondary raw materials. Their high lignin and cellulose content, combined with intrinsic hardness and low cost, make them attractive as reinforcing fillers for thermoplastic composites [29,30].

However, despite the advantages, composites based on recycled polymers and natural fillers present specific challenges: (i) there is a chemical incompatibility between the non-polar nature of polyolefin matrices and the strongly polar surface of lignocellulosic fillers, which often leads to poor interfacial adhesion, void formation, and micro-delamination [31,32]; (ii) polymer degradation during previous processing and service life can produce chain scission and oxidation in the recycled polymer, lowering molecular weight and further limiting stress transfer from matrix to filler [33]; (iii) achieving a homogeneous dispersion of particles is also more difficult in heterogeneous systems, and the presence of the filler tends to increase brittleness and processing defects may occur [34]; (iv) long-term durability may be compromised by moisture uptake or thermal aging, which can worsen interfacial weakness [35].

To overcome these limitations, coupling agents are commonly employed to promote chemical and physical interactions between the hydrophobic matrix and the hydrophilic filler surface [36,37]. Compatibilizers based on maleic-anhydride-grafted polypropylene (PPC), for example, can be employed for this goal. The presence of maleic anhydride groups, in fact, enables the formation of covalent or hydrogen bonds with the hydroxyl functionalities of the lignocellulosic filler, thereby improving interfacial bonding and enhancing both mechanical performance and long-term stability [38,39].

The present work explores the production of sustainable composites based on recycled polypropylene filled with hazelnut shell powder (HS), with and without maleic-anhydride-grafted polypropylene coupling agent. The investigation aims to assess whether the combined use of an agro-industrial by-product and a compatibilizer can restore or even improve the mechanical performance of RPP, enabling its use in applications that require mechanical reliability such as irrigation fittings. In addition to mechanical characterization, this study includes a detailed rheological and morphological analysis to elucidate the effect of filler addition and compatibilization on melt flow and interfacial adhesion. By demonstrating that hazelnut shell flour can be effectively integrated as a functional filler in recycled polypropylene, this study highlights a scalable strategy to merge plastic recycling with agricultural waste valorization, thereby supporting the transition toward a circular economy.

Beyond the numerous studies describing the formulation and characterization of natural filler-reinforced polyolefin composites, very few works extend the analysis to the validation of these materials on a real industrial scale. In contrast, the present study not only investigates the rheological, morphological, and mechanical behavior of RPP and hazelnut shell composites, but also brings them to the level of industrial implementation. Full-size irrigation fittings were injection-molded using standard processing equipment and tested under hydraulic pressure in an operational industrial facility. This approach allows us to directly correlate material properties with the functional performance of a real device, an aspect rarely addressed in composite research but essential for establishing the practical applicability of sustainable materials.

## 2. Materials and Methods

### 2.1. Materials

Virgin and recycled polypropylene samples used in this work were provided by Irritec Spa (Messina, Italy) and their description is reported in Table 1. The hazelnut shells (HSs) came from crops in the Nebrodi Mountains area (Messina, Italy) and were kindly supplied by the Damiano Company (Messina, Italy). Hazelnut shells typically contain approximately 25–32 wt% cellulose, 20–30 wt% hemicellulose, and 35–45 wt% lignin, with a minor ash fraction (1–3 wt%) [40,41]. Maleic-anhydride-functionalized polypropylene, known by the commercial code TECNOBOND PP/C, containing 0.8–1.0 wt% maleic anhydride, was provided by Tecnofilm Spa (Fermo, Italy) and used as the coupling agent.

The RPP was a commercial-grade polymer obtained by the recycling of post-consumer materials. As is typical of post-consumer materials, the exact number of previous recycling cycles and the possible presence of residual additives were not specified by the supplier. No additional additives were introduced during this work.

### 2.2. Preparation of Filler and Composites

The hazelnut shells were ground using an electric grinder (Moulinex, Écully, France), sieved, and the fraction with particle size below 500 µm was collected for further processing. Because the polyolefin matrix is non-polar whereas the lignocellulosic filler is intrinsically polar, poor interfacial adhesion is expected. In addition, traces of contaminants may be present in the recycled polypropylene. To enhance the compatibility between the two phases and mitigate possible interfacial defects, 5 wt% of maleic-anhydride-grafted polypropylene was incorporated as a coupling agent. It should be noted that the HS powder was used without any surface treatment or chemical modification. The only functionalized component in the formulations is the PPC, which acts as a compatibilizer. No grafting or surface functionalization of the filler was performed.

The composite formulations were prepared using an internal mixer (Plasticorder, Brabender, Duisburg, Germany) at 210 °C and 60 rpm for approximately 5 min, which was sufficient to reach a steady-state torque. The obtained formulations were then extruded and pelletized in a twin-screw compounder. The sample codes and the composition of the investigated composites are reported in Table 2.

Virgin and recycled polypropylene samples were processed under the same conditions.

Samples for the mechanical characterization were produced by compression molding using a laboratory press (Carver, Wabash, IN, USA) at 210 °C.

Preliminary industrial trials were also performed to injection-mold sleeve bodies and their corresponding nuts from the composites RPP/HS5 and RPP/HS5/PPC, as well as from the reference PP sample. The tree samples were injection-molded on a press (Zhafir ZE 1500 III, 150 t, Ebermannsdorf, Germany) under the processing conditions summarized in Table 3.

For the industrial preparation of the composites, the obtained formulations were extruded and pelletized using a high-volume co-rotating twin-screw compounder. Specifically, compounding was carried out on a COMAC EBC 30HT/37D (Cerro Maggiore, Italy) extruder, equipped with 32 mm diameter screws (L/D = 37:1), two-lobe geometry, and an intermeshing coefficient of 1.625. The temperature profile was set to 170-190-200-200-200-200-200 °C, while the die head was maintained at 240 °C, with a melt pressure of 95 bar. The screw speed during processing was 200 rpm, with a steady-state throughput of approximately 25 kg/h. Palletization was performed by hot-face cutting (cutter speed 1600 rpm), followed by cooling in a water bath at 28 °C (flow rate 4.8 m^3^/h).

### 2.3. Thermogravimetric Analysis

Thermogravimetric analysis (TGA) was carried out to define the thermal stability of hazelnut shells. The analysis was carried out by using a TGA 8000 instrument (PerkinElmer, Waltham, MA, USA). HS samples were placed in an alumina pan and heated under nitrogen atmosphere (flow rate: 30 mL/min) from 40 °C to 950 °C at a constant heating rate of 20 °C/min. After reaching 950 °C, an isothermal step of 15 min was applied under oxygen flow (30 mL/min).

### 2.4. Morphological Analysis

The morphology of the HS powder and composite formulations were observed using a scanning electron microscope (Phenom ProX, Phenom-World, Eindhoven, The Netherlands) with an optical magnification range of 20–135×, an electron magnification range of 80–1.3 × 10^5^, a maximal digital zoom of 12×, and acceleration voltages of 15 kV. SEM observations were carried out using the backscattered electron detector (BSD). No sputter coating was applied to the samples. For cross-sectional SEM analysis, the composites were immersed in liquid nitrogen and then cryo-fractured.

The distribution of particle equivalent diameters was assessed using the ImageJ v1.54g software and the relative the DiameterJ v1.018 plugin. The diameters of 100 particles for each SEM image were measured. Each measurement was performed in triplicate.

### 2.5. Rheological Characterization

Rheological properties of the samples were analyzed, using a rotational rheometer (ARES- G2, TA Instruments, New Castle, DE, USA) equipped with a 25 mm parallel-plate geometry with a plate gap of 1.00 mm. Before each frequency sweep, the instrument conducted an automatic stabilization step, during which the sample was equilibrated until torque and normal force achieved steady values, ensuring reproducible testing conditions. All the tests were performed at 210 °C, in frequency sweep mode in the range 0.1–100 rad/s, by imposing a constant stress of 1 Pa, according to standard rheological protocols for molten polyolefins. The non-isothermal tests were conducted with a capillary viscometer (Rheologic 1000, CEAST, Torino, Italy) equipped with a tensile module and operated at the same temperature.

### 2.6. FTIR Analysis

Chemical and structural characterizations of the sample surfaces were assessed by FT-IR/ATR analysis, carried out using a Perkin-Elmer FT-IR/NIR Spectrum 400 spectrophotometer (Shelton, CT, USA). The absorbance spectra were recorded in the wavenumber range 4000–400 cm^−1^.

### 2.7. Mechanical Characterization

The tensile behavior was evaluated on five replicate specimens for each formulation using a universal testing machine (Instron, model 3365, Norwood, MA, USA). A 1 kN load cell was used. Rectangular samples (approximately 60 mm × 10 mm) were tested after measuring their thickness. A two-stage crosshead speed was applied: 1 mm min^−1^ for the initial 2 min, followed by 50 mm min^−1^ until fracture. The gauge length was set to 30 mm. Mean values and standard deviations were calculated for the elastic modulus (E), tensile strength (TS), and elongation at break (EB).

### 2.8. Internal Pressure Test

The printed composite fittings were tested for internal pressure resistance following an internal procedure. In more detail, the fitting connects two pipes and holds the pipe with a nut. The nut keeps the pipe between itself and the body, when the user forces it on the pipe. One of the pipes is plugged at the end while the other pipe is connected to a pumping system that pushes the water from 0 up to 3.50 bar and all through the system, so the fitting and the two pipes are under pressure. The result of the test will be positive if the fitting is able to hold both pipes with any leakage, and the fittings do not suffer any damage inside the pressure range. For each formulation, at least five fittings were tested under the internal pressure protocol described above.

The fittings produced in this study are intended for thin-wall seasonal drip irrigation lines, whose operational pressure typically ranges between 0.5 and 1.8 bar. Therefore, the hydraulic test was performed up to 3.5 bar, corresponding to more than twice the maximum operating pressure.

## 3. Results and Discussion

### 3.1. Preparation and Characterization of HS Powder

**Figure 1:**
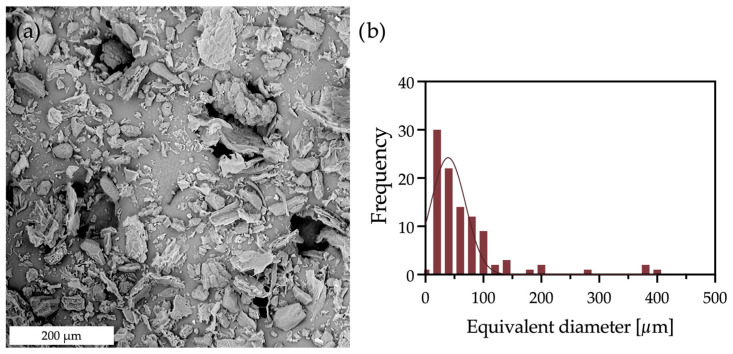
SEM micrograph of the hazelnut shell particles (**a**) after the milling and sieving process, with the corresponding particle size distribution (**b**).

Hazelnut shells were successfully ground and sieved to obtain a fine powder with a particle size below 500 µm. This size fraction was selected to ensure a homogeneous dispersion within the polymer matrix and to limit the presence of large particles that could negatively affect the mechanical properties of the composites. A representative SEM micrograph of the HS powder is shown in Figure 1, together with the corresponding particle size distribution (Figure 1b).

The SEM micrograph (Figure 1a) highlights the irregular shape of the particles, which is typical of lignocellulosic materials obtained by mechanical grinding. The particle size distribution (Figure 1b) confirms that all particles are below 500 µm, in agreement with the sieving step adopted during filler preparation. The distribution is mainly concentrated in the smaller size range, with only a limited fraction of coarser particles, with an average equivalent diameter of about 50 µm. Such morphology and size distribution are expected to promote a relatively homogeneous dispersion in the virgin and recycled polypropylene matrix.

The obtained powder was subsequently melt-mixed with the virgin and recycled polymeric matrices, with or without the coupling agent, to produce the different composites, as detailed in Table 2.

### 3.2. Thermogravimetric Analysis

Thermogravimetric analysis (TGA) was performed to verify hazelnut shell powder thermal stability under the processing conditions adopted. Figure S1 reports the TGA and DTG curves of the hazelnut flour under nitrogen atmosphere. The first major degradation event begins at an onset temperature of 280 °C, followed by a second decomposition stage at 392 °C. These two degradation steps are consistent with the typical thermal behavior of lignocellulosic biomass, in which hemicellulose and cellulose decompose first, followed by the slower degradation of lignin. No significant mass loss is detected near the processing temperature of 210 °C, confirming that the hazelnut shell powder is thermally stable during melt compounding. These results validate its suitability as a filler for PP-based composites processed under conventional extrusion and injection-molding conditions.

### 3.3. Characterization of the Composite Formulations

**Figure 2:**
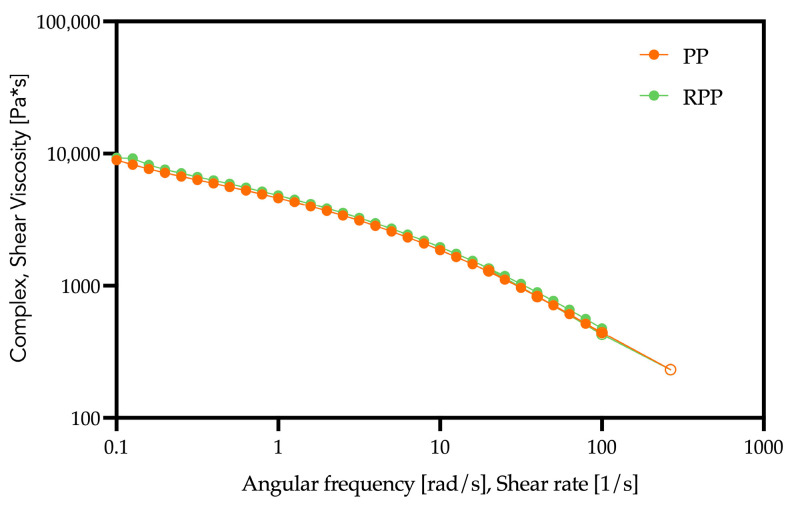
Flow curves of the two matrices: PP and RPP. Closed symbols refer to the rotational rheometer, open symbols to the capillary viscometer.

The resulting formulations were extruded to obtain homogeneous pellets, and their rheological behaviors were then evaluated in oscillatory shear mode; the relative results are reported in Figure 2.

Both PP and RPP exhibit the typical shear-thinning trend of polyolefin melts, with complex viscosity decreasing as angular frequency (or shear rate) increases. The data obtained from the rotational rheometer and from the capillary viscometer overlap well, especially for PP, confirming that both matrices follow the Cox–Merz rule. Moreover, the recycled polypropylene shows complex viscosity values very close to those of the virgin PP, indicating that the recycling process did not significantly alter the melt-flow behavior of the polymer. However, the rheological behavior of the recycled polypropylene should not be interpreted as the viscosity of the same polymer after a recycling cycle, since the RPP used in this study is a commercial post-consumer grade provided by a different manufacturer. Its melt-flow properties may reflect the original formulation and the presence of residual stabilizers or additives, which can contribute to a viscosity profile comparable to that of the virgin PP. Therefore, the similarity between PP and RPP in complex viscosity does not necessarily reflect the effect of recycling, but rather the intrinsic characteristics of the specific commercial materials employed.

**Figure 3:**
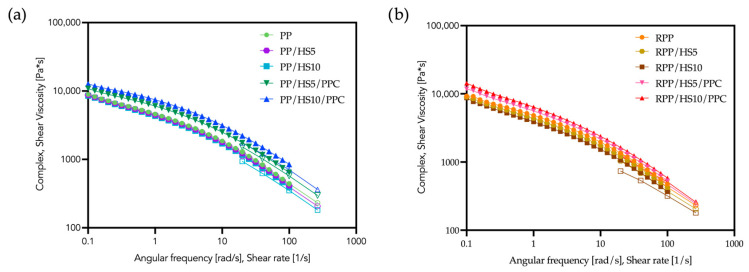
Flow curves of the PP composites (**a**) and RPP composites (**b**). Closed symbols refer to the rotational rheometer, open symbols to the capillary viscometer.

To assess how the incorporation of hazelnut shell flour and the coupling agent affects melt-flow behavior of the polymers, complex-viscosity flow curves were determined by rotational rheometer and capillary viscometry. The results are shown in Figure 3. In all the formulations, the addition of 5 wt% of hazelnut shell powder produces smaller variation in viscosity with respect to the 10 wt% added ones, as logically expected.

The viscosity curves obtained by the rotational rheometer and by the capillary viscometry do not perfectly overlap for any of the composite formulations, indicating that these materials do not follow the Cox–Merz rule. This deviation is consistent with previous findings reported in the scientific literature which show that the Cox–Merz relationship generally holds for homogeneous polymer melts but often fails in heterogeneous and multiphase systems [42].

Composites produced without the PPC show a slight reduction in complex viscosity values only at intermediate and high shear, whereas at low shear rates the viscosity remains comparable to that of PP and RPP. Therefore, the effect of the filler alone should be considered minimal and strongly dependent on the deformation regime. When the compatibilizer is incorporated, the viscosity curves shift above the corresponding matrix, as generally expected when solid particles are dispersed in a polymer melt. The viscosity value observed in the uncompatibilized systems could be related to the very poor interfacial adhesion between the polyolefin matrix, both PP and RPP, and the hazelnut shell flour. On the other hand, the addition of PPC is expected to promote chemical and physical interactions between the non-polar matrix and the polar lignocellulosic filler, giving rise to an increase in complex viscosity [43].

At high shear rates, however, the viscosity curves of matrices and composites tend to converge, and the difference between the neat polymers and the filled systems becomes negligible. Such behavior suggests that the processing parameters commonly used for the injection-molding of the neat matrices could be applied to these composites without significant modifications.

**Figure 4:**
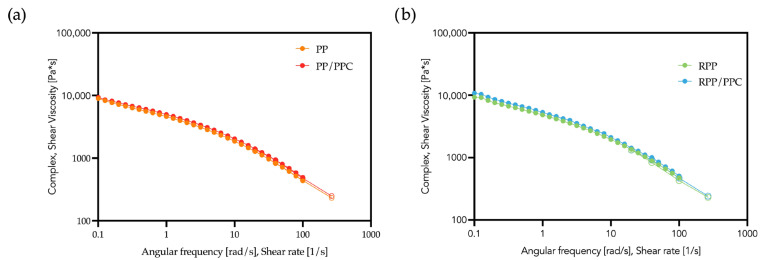
Flow curves of PP/PPC (**a**) and RPP/PPC (**b**). Pure matrices are reported again for comparison. Closed symbols refer to the rotational rheometer, open symbols to the capillary viscometer.

Additional measurements were performed on PP and RPP containing PPC but no hazelnut shell powder for comparison. As shown in Figure 4, the presence of the compatibilizer causes a very moderate increase in complex viscosity for both matrices. The effect is significantly smaller if compared with the filled systems, confirming that the strong viscosity enhancement observed in the composites arises from the combined contribution of PPC and HS particles.

**Figure 5:**
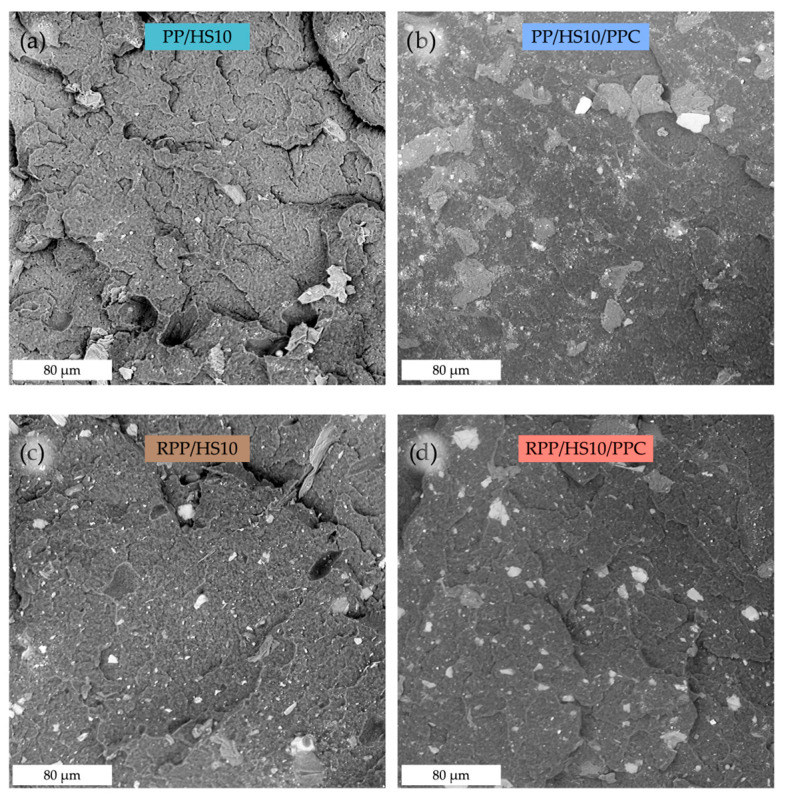
SEM micrographs of the cross-sectional surfaces of the cryo-fracture PP/HS10 (**a**), PP/HS10/PPC (**b**), RPP/HS10 (**c**), and RPP/HS10/PPC (**d**) composites.

The abovementioned rheological behaviors were confirmed by morphological analysis performed on the cross-sectional surface of the cryo-fracture composites, as shown in Figure 4. As already observed in similar systems [38,44], SEM micrographs of the uncompatibilized composites (Figure 5a,c) highlight the presence of voids at the filler–matrix interface, confirming the lack of effective bonding between the two phases.

Conversely, as clearly observed in Figure 5b,d, the presence of the coupling agent markedly improves the interfacial adhesion between the PP and RPP matrices and the hazelnut shell powder. In the composites without compatibilizer, voids and gaps are visible at the matrix–filler interface, indicating poor bonding and limited stress transfer. The compatibilized samples displayed better adhesion between the two phases, with the filler particles well embedded in the polymer matrix. This morphological evidence confirms the role of PPC in promoting effective chemical and physical interactions, thereby enhancing the overall integrity of the composite structure.

**Figure 6:**
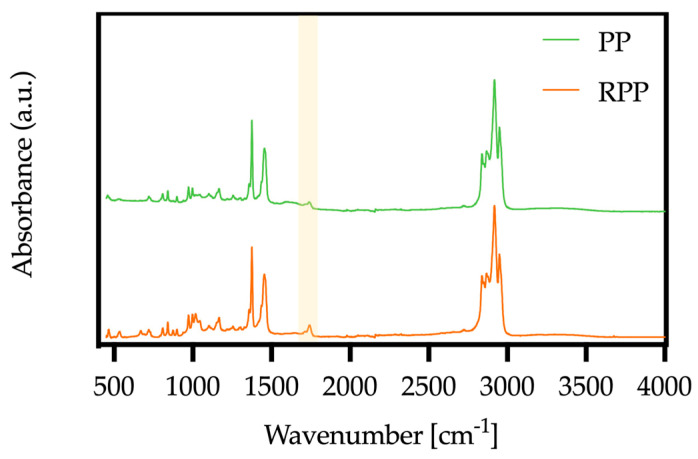
FTIR spectra of PP and RPP samples. Absorption band at 1735 cm^−1^ is highlighted.

This effect is particularly pronounced in the composites based on recycled polypropylene (RPP; see Figure 3b and Figure 5d). In this case, the recycled matrix contains oxygenated groups, as evidenced by the FTIR absorption band at 1735 cm^−1^ (Figure 6, see the highlighted section), which favor additional interactions with the maleic-anhydride functionalities of PPC. The combined presence of these oxygenated species and the coupling agent therefore enhances the interfacial adhesion and amplifies the viscosity increase observed for the RPP composites.

The role of PPC can be explained by the reactivity of the anhydride groups grafted along the PP backbone. These functional groups are highly susceptible to ring-opening reactions at the typical melt-processing temperatures (200–210 °C) [45,46]. In the presence of hydroxyl groups, such as those abundant on the lignocellulosic hazelnut shell surface, the anhydride groups undergo esterification, forming covalent ester bonds at the filler–matrix interface [43,44]. This mechanism explains the improved interfacial adhesion observed in the compatibilized composites. In addition, recycled polypropylene itself plays an active role. Because of repeated processing and oxidative degradation, RPP contains oxygenated species such as carbonyl and hydroxyl groups, as confirmed by FTIR analysis. These polar groups can react with the anhydride moieties of PPC, leading to additional ester linkages within the polymer phase [47]. This dual reactivity, towards both the filler and the recycled matrix, explains the stronger compatibilization effect observed in RPP composites compared to those based on virgin PP, where such oxygenated groups are absent. These reaction mechanisms can explain why compatibilized RPP-based composites exhibit enhanced viscosity and reduced void formation compared with their uncompatibilized counterparts and even with compatibilized virgin PP composites.

### 3.4. Mechanical Characterization

The above characterized pellets were then processed into specimens by compression molding, and their mechanical performance was evaluated by tensile tests to determine the elastic modulus (E), tensile strength (TS), and elongation at break (EB) of both the neat matrices and the corresponding composites. The results are reported in Table 4.

The incorporation of hazelnut shell powder increases the stiffness of both virgin and recycled polypropylene (see Table 4). In more detail, the elastic modulus (E) rises progressively with filler loading, reaching values close to 1.0 and 1.2 GPa, respectively, for the PP and RPP composites containing 10 wt% of HS. Consequently, the elongation at break (EB) decreases sharply already at 5 wt % filler and becomes particularly low (about 6%) at 10 wt % of HS incorporated. The marked reduction in EB is accompanied by a decrease in tensile strength (TS), reflecting the embrittlement typically induced by rigid lignocellulosic fillers and the weak filler–matrix interface when no coupling agent is present. The addition of PPC significantly mitigates these effects. In both PP- and RPP-based composites, in fact, the coupling agent leads to a less marked decrease in EB and TS, while causing only minor changes in E. This behavior confirms that PPC improves the interfacial adhesion between the polyolefin matrix and the polar hazelnut shell particles, promoting more efficient stress transfer. The beneficial role of the coupling agent is slightly more pronounced in the recycled polypropylene composites, where the oxygenated species (detected by FTIR analysis; see Figure 6) likely promote additional interactions with the maleic-anhydride functionalities, further strengthening the filler–matrix bonding. These mechanical results are consistent with the rheological findings, where the compatibilized systems exhibited higher complex viscosity (Figure 3), and with the morphological evidence provided by SEM. In particular, the micrographs (Figure 5b,d) show the absence of interfacial voids in the presence of PPC, in contrast with the poor adhesion and void formation observed in the uncompatibilized composites.

In summary, the incorporation of hazelnut shell powder together with the coupling agent leads to a slight increase in elastic modulus while reducing the elongation at break. Nevertheless, this reduction in ductility is of limited relevance for the intended application, since molded components such as irrigation fittings do not require high elongation to ensure adequate performance. The mechanical performance of the composites remains fully adequate for irrigation fittings.

### 3.5. Injection Molded Samples and Hydraulic Test

**Figure 7:**
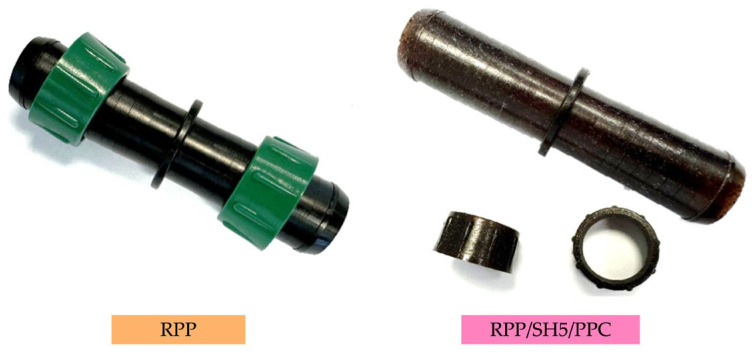
Injection-molded irrigation fittings produced with recycled polypropylene (RPP, (**left**)) and with the compatibilized green composite RPP/HS5/PPC (**right**).

Preliminary industrial trials were carried out by injection-molding sleeve bodies and their corresponding nuts using RPP, RPP/HS5, and RPP/HS5/PPC. Examples of fittings produced with the traditional RPP and RPP/HS5/PPC composite are shown in Figure 7.

According to the procedure reported in Section 2.7 of Materials and Methods, the fittings were subjected to an internal pressure test in which water pressure was gradually increased from 0 up to 3.5 bar, so that both the fitting and the connected pipes were continuously stressed. The test was considered successful when the fitting maintained a leak-free seal and showed no visible damage throughout the applied pressure range. All the samples satisfied these criteria, demonstrating their ability to withstand water pressures higher than 3.5 bar without leakage or structural failure and thus confirming performance comparable to fittings produced from virgin PP.

These results confirm the suitability of the composites, especially the compatibilized formulations, for the production of irrigation fittings, validating their potential for real industrial applications. The fittings produced in this study are intended for thin-wall seasonal drip irrigation lines, whose operational pressure typically ranges between 0.5 and 1.8 bar.

## 4. Conclusions

Developing irrigation components from eco-sustainable materials that match the performance of virgin fossil-based polymers is an essential step toward reducing the environmental impact of water management systems. In this work, hazelnut shell flour was incorporated into both virgin and recycled polypropylene to produce sustainable composites suitable for injection-molded fittings. Because of the intrinsic incompatibility between the non-polar matrix and the polar filler, a maleic-anhydride-grafted polypropylene was used as a coupling agent. Rheological measurements confirmed that the recycled matrix retained processability comparable to that of virgin PP. The presence of the compatibilizer increased the complex viscosity of the composites (particularly for RPP) consistent with the oxygenated groups observed by FT-IR and the improved filler–matrix adhesion highlighted by SEM. Overall, the rheological results clearly indicate that the composites exhibit a processability similar to that of pure polypropylene, a finding further corroborated by preliminary industrial injection-molding trials in which the fittings were easily produced. The addition of 10 wt% hazelnut shell powder raised the elastic modulus from about 930 MPa in neat PP to approximately 1.0 GPa and up to around 1.25 GPa in the recycled matrix, demonstrating a clear stiffening effect. As expected, the elongation at break decreased from 65% of neat PP to about 6% in the uncompatibilized RPP/HS10 composite. The coupling agent partly mitigated this loss, allowing EB values to recover to 18% in RPP/HS10/PPC. Tensile strength followed the same trend, with the compatibilizer enabling a partial recovery compared with the uncompatibilized systems. Tensile tests showed a moderate increase in elastic modulus but a reduction in elongation at break after HS addition. The coupling agent mitigated this effect and partially restored tensile properties. Preliminary industrial trials demonstrated that fittings injection-molded from RPP/HS5 and RPP/HS5/PPC composites resisted internal water pressures above 3.5 bar without leakage or structural damage. In addition, long-term durability and environmental aging tests in full field conditions are currently underway to assess the performance of the RPP/HS composites under real irrigation environments. These results, which include exposure to UV radiation, temperature fluctuations, and hydraulic cycling, will be reported in a forthcoming study dedicated to long-term behavior.

The combination of RPP with an agro-industrial residue of negligible cost represents a viable route toward materials with reduced environmental footprint. This approach enables the valorization of waste streams while maintaining performance levels suitable for industrial irrigation components.

Overall, combining recycled polypropylene with hazelnut shell flour and an appropriate compatibilizer offers a scalable route to high-performance, low-impact composites, confirming their suitability for real irrigation applications and meeting the need for sustainable alternatives to conventional virgin polyolefin fittings.

## Figures and Tables

**Table 1:** Description of virgin and recycled polypropylene samples used in this work.

Polymer	Sample Code	Commercial Name/Manufacturer
Virgin Polypropylene	PP	Capilene/Carmel Olefins (Haifa, Israel)
Recycled Polypropylene	RPP	Mafill CR 2044 BK/Ravago (Bergamo, Italy)

**Table 2:** Sample code name and relative composition of the investigated composites and pure matrices.

Sample Code	Polymer	HS [wt%]	PPC [wt%]
PP	PP	0	0
PP/HS5	PP	5	0
PP/HS10	PP	10	0
PP/HS5/PPC	PP	5	5
PP/HS10/PPC	PP	10	5
RPP	RPP	0	0
RPP/HS5	RPP	5	0
RPP/HS10	RPP	10	0
RPP/HS5/PPC	RPP	5	5
RPP/HS10/PPC	RPP	10	5

**Table 3:** Processing parameters adopted during the injection-molding of sleeve bodies and nuts from RPP/N5, RPP/N5/PPC composites and the reference PP sample.

Parameter	Value
Nozzle temperature	225 °C
Hot-runner temperature	225 °C
Mold temperature	16 °C
Back pressure	100 bar
Maximum injection pressure	1200 bar
Holding pressure	600 bar
Cycle time	22 s

**Table 4:** Elastic modulus (E), tensile strength (TS), and elongation at break (EB) of both the neat matrices and the corresponding composites.

Sample	E [MPa]	TS [MPa]	EB [%]
PP	930 ± 5	23 ± 2	65 ± 5
PP/HS5	973 ± 4	19 ± 1	12 ± 3
PP/HS10	1006 ± 11	12 ± 3	5.6 ± 0.6
PP/HS5/PPC	1001 ± 13	21 ± 3	34 ± 5
PP/HS10/PPC	1050 ± 12	16 ± 1	16 ± 2
RPP	960 ± 8	20 ± 1	63 ± 4
RPP/HS5	1086 ± 12	18 ± 3	14 ± 4
RPP/HS10	1200 ± 16	12 ± 2	6.2 ± 0.8
RPP/HS5/PPC	1104 ± 12	20 ± 6	43 ± 6
RPP/HS10/PPC	1250 ± 14	18 ± 5	18 ± 4

## Data Availability

The data presented in this study are available on reasonable request from the corresponding author.

## Supplementary Materials

The following supporting information can be downloaded at: https://www.mdpi.com/article/10.3390/polym17233207/s1, Figure S1: Thermogravimetric (TGA) analysis of the hazelnut shell powder.
