# Supplementary material for: Sustainable Composites from Recycled Polypropylene and Hazelnut Shell Flour for Application in Irrigation Systems
Source: Polymers (Basel). 2025 Dec 1;17(23):3207. doi: 10.3390/polym17233207 (PMC12694308; doi:10.3390/polym17233207)
Supplement: Supplementary file 1 [file polymers-17-03207-s001.zip › polymers-3947677-supplementary.pdf]

Supporting Materials

Sustainable Composites from Recycled Polypropylene and Hazelnut Shell Flour for Application in Irrigation Systems

Francesco Paolo La Mantia [1,2,*], Roberto Scaffaro [1,2,*], Giuseppe Balsamo [1], Carmelo Giuffré [3], Erica Gea Rodi [3], Simone Corviseri [3] and Maria Clara Citarrella [1,2]

[1] INSTM—Consortium for Materials Science and Technology, Via Giusti 9, 50125 Florence, FI, Italy; giuseppe.balsamo01@communityunipa.it (G.B.); mariaclara.citarrella@unipa.it (M.C.C.)

[2] Department of Engineering, University of Palermo, Viale Delle Scienze, ed. 6, 90128 Palermo, PA, Italy

[3] Irritec S.p.A., Via Gambitta Conforto, 98071 Capo d'Orlando, ME, Italy; carmelo.giuffre@irritec.com (C.G.); erica.rodi@irritec.com (E.G.R.); simone.corviseri@irritec.com (S.C.)

* Correspondence: francescopaolo.lamantia@unipa.it (F.P.L.M.); roberto.scaffaro@unipa.it (R.S.)

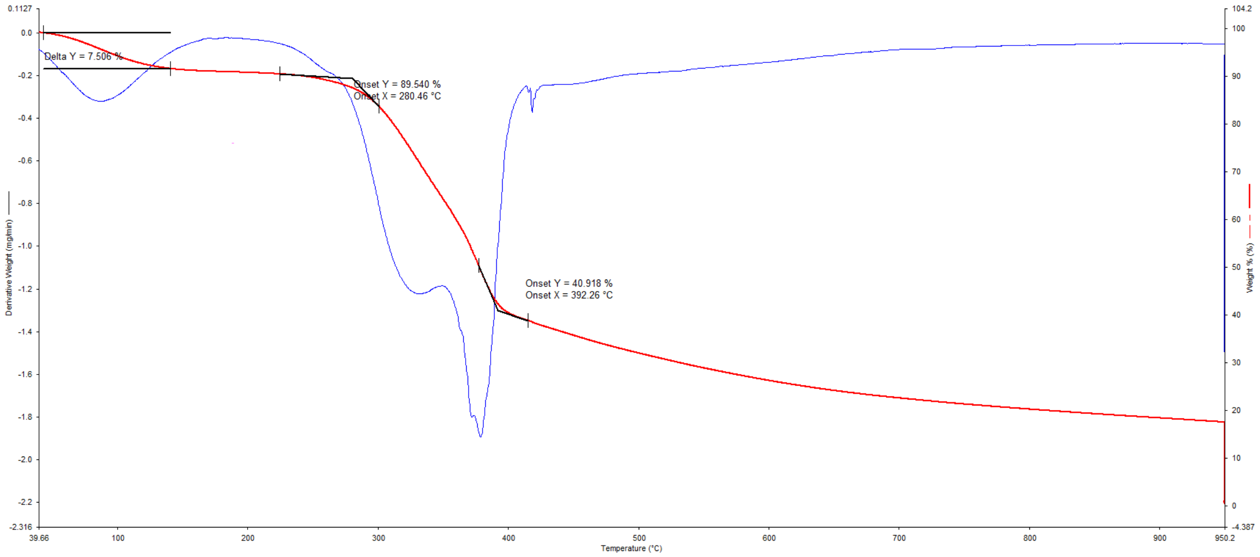

Figure S1. Thermogravimetric (TGA) analysis of the hazelnut shell powder.
